# SECONDARY HYPERPARATHYROIDISM AFTER BARIATRIC SURGERY: TREATMENT IS WITH CALCIUM CARBONATE OR CALCIUM CITRATE?

**DOI:** 10.1590/S0102-6720201500S100013

**Published:** 2015-12

**Authors:** Giorgio Alfredo Pedroso BARETTA, Maria Paula Carlini CAMBI, Arieli Luz RODRIGUES, Silvana Aparecida MENDES

**Affiliations:** Vita Batel Hospital, Curitiba, PR, Brazil

**Keywords:** Secondary hyperparathyroidism, Bariatric surgery, Nutritional deficiencies

## Abstract

***Background*:**

Bariatric surgery, especially Roux-en-Y gastric bypass, can cause serious nutritional complications arising from poor absorption of essential nutrients. Secondary hyperparathyroidism is one such complications that leads to increased parathyroid hormone levels due to a decrease in calcium and vitamin D, which may compromise bone health.

**Aim:**

: To compare calcium carbonate and calcium citrate in the treatment of secondary hyperparathyroidism.

**Method:**

: Patients were selected on the basis of their abnormal biochemical test and treatment was randomly done with citrate or calcium carbonate.

**Results:**

: After 60 days of supplementation, biochemical tests were repeated, showing improvement in both groups.

**Conclusion:**

: Supplementation with calcium (citrate or carbonate) and vitamin D is recommended after surgery for prevention of secondary hyperparathyroidism.

## Abstract (pt)

***Racional*:**

A cirurgia bariátrica, especialmente a gastroplastia em Y-de-Roux, pode causar complicações nutricionais importantes que derivam da má absorção de nutrientes essenciais. O hiperparatireoidismo secundário é uma delas que cursa com o aumento do hormônio da paratireoide e consequente diminuição de cálcio e vitamina D, o que pode comprometer a saúde óssea.

**Objetivo:**

: Comparar o tratamento do hiperparatireoidismo secundário com o uso de carbonato e citrato de cálcio.

**Métodos:**

: Os pacientes foram selecionados a partir de seus exames bioquímicos alterados e o tratamento foi sugerido aleatoriamente com citrato ou carbonato de cálcio.

**Resultados:**

: Após 60 dias de suplementação foram reavaliados os exames bioquímicos e percebeu-se melhora em ambos os grupos, tanto com citrato como com carbonato de cálcio.

**Conclusão:**

: Recomenda-se o uso de suplementação de cálcio (citrato ou carbonato) e vitamina D após a operação para prevenção do hiperparatireoidismo secundário.

## INTRODUCTION

Secondary hyperparathyroidism is a relatively common nutritional disorder among patients undergoing bariatric surgery, in its various techniques. The disease secondary to hyperparathyroidism is characterized by a negative calcium balance, associated or not with vitamin D, which causes an abrupt increase in parathyroid hormone (PTH) levels, with consequent osteopenia or osteoporosis. 

The changes in bone metabolism after Roux-en-Y gastric bypass (RYGB) are related to the alterations in intestinal absorption of various nutrients, besides a lower intake of protein foods and hindered absorption of vitamin D.

This study had as objective to analyze the treatment of secondary hyperparathyroidism after RYGB with different calcium salts.

## METHODS

The study was approved by the Research Ethics Committee of Hospital Vita Batel, and all participants signed an informed consent form for inclusion in the study. It was prospective, randomized with 20 patients undergoing RYGB at a private hospital in Curitiba, Paraná, Brazil, in 2013-2014. 

Patients with chronic diseases or using medications that interfere with bone metabolism were excluded. 

Patients were assessed in regard to the following data: age, gender, body mass index, measurement of serum calcium, alkaline phosphatase, vitamin D and PTH. Patients were selected on basis of their levels of alkaline phosphatase, vitamin D and PTH, which were mainly abnormal. At this time, randomized treatment was suggested for the patient with calcium citrate or calcium carbonate to form a group of 10 patients for each treatment. Group 1 received 600 mg calcium citrate, together with 400 IU vitamin D, twice daily for 60 days. Group 2 received 600 mg calcium carbonate combined with 400 IU vitamin D, twice daily. Both groups received the supplements as tablets, and participants were instructed to take them with water and not with meals containing iron.

All patients underwent bone densitometry examinations, and they brought their laboratory test results. Everyone was asked if they exercised and how often.

The data were tabulated in Excel and statistical analysis was performed using tables containing the analysis of the variables: age, body mass index, and serum calcium, alkaline phosphatase, PTH and vitamin D, with the initial (before treatment) and final (after treatment of 60 days) measurements. The Mann-Whitney test was used, and the significance level adopted was α<0.05.

## RESULTS

Of the patients selected, nine were men and 11 women. In group 1 treated with calcium carbonate, there were six men and four women, and in group 2 treated with calcium citrate, there were three men and seven women. There was no statistical significance in gender. In group 1, all patients were sedentary, without any physical activity program. In group 2, four women worked out twice a week with weights training and treadmill. All others were sedentary.

Bone densitometry of the 20 participants was normal for femur and spine. No statistically significant difference was observed between the groups in relation to bone mineral density of the lumbar spine and femoral neck. 

The medium age was 46 years old in both groups (Table 1).

**TABLE 1:** - Statistical analysis between calcium carbonate (citracal) and calcium citrate (compounded) with regard to age

**Supplement**	**n**	**AGE**	**Mann-Whitney test**			
		mín - max	Mean	±	SD	p
Calcium carbonate	10	25-68	49.5	±	14.4	0.16
Calcium citrate	10	25-55	42.7	±	10.8	

n= number of patients; SD=standard deviation; p=probability value

**TABLE 2:** - Statistical analysis between IMC and calcium carbonate (citracal) and calcium citrate (compounded)

**Supplement**	**n**	**IMC**	**Mann-Whitney test**			
		min - max	Mean	±	SD	p
Calcium carbonate	10	26,0 - 39,4	33,0	±	4,3	0,97
Calcium citrate	10	29,5 - 36,0	32,8	±	2,4	

n=number of patients; min-max= minimum and maximum values; SD=standard deviation; p=probability value

According to the Tables, no statistically significant differences were found between the two calcium salts, where they were both equally effective in correcting secondary hyperparathyroidism.

**TABLE 3:** - Statistical analysis between calcium carbonate (citracal) and calcium citrate (compounded) regarding to biochemical variables

**Supplement**		**Variable**	**Mann-Whitney test**			
	**n**	**Min-Max**	**Mean**	**±**	**SD**	**p**
		Calcium 1				
Calcium carbonate	10	8,1 - 8,9	8.7	±	0.2	0.97
Calcium citrate	10	8,2 - 9,0	8.6	±	0.3	
		Alkaline phosphatase 1				
Calcium carbonate	10	119,4 - 180	135.8	±	18.6	0.91
Calcium citrate	10	100,0 - 163,0	132.8	±	23.2	
		PTH 1				
Calcium carbonate	10	68,7 - 92,0	82.7	±	8.8	0.80
Calcium citrate	10	67,5 - 95,0	84.0	±	8.9	
		Vitamin D 1				
Calcium carbonate	10	17,0 - 28,9	-22.8	±	4.4	0.85
Calcium citrate	10	16,0 - 29,0	22.2	±	3.9	
		Calcium 2				
Calcium carbonate	10	8,1 - 8,9	8.7	±	0.3	0.48
Calcium citrate	10	8,1 - 10,0	8.6	±	0.6	
		Alkaline phosphatase 2				
Calcium carbonate	10	82,3 - 120,0	98.3	±	12.5	0.58
Calcium citrate	10	87,5 - 120,0	101.0	±	10.4	
		PTH 2				
Calcium carbonate	10	29,0 - 65,0	47.9	±	9.7	0.12
Calcium citrate	10	42,0 - 64,0	54.2	±	7.2	
		Vitamin D 2				
Calcium carbonate	10	25,0 - 35,0	30.6	±	3.3	0.85
Calcium citrate	10	25,0 - 36,0	30.9	±	3.6	

n=number of patients; min - max - minimum and maximum values; SD= standard deviation; p=probability value

## DISCUSSION

RYGB causes a significant reduction in the absorption of nutrients such as calcium and vitamin D^1^^,^ 2. This nutritional loss is easily detected through regular metabolic monitoring. The mineral density is low independent the lost weight after RYGB for low calcium absorption cause low vitamin D activation dependent the calcium^3^.

This study revealed the presence of secondary hyperparathyroidism after RYGB. All patients subjected to surgical treatment for obesity must use calcium and vitamin D supplements to prevent possible deficiencies. The main question has been what kind of supplements would be most effective in protecting the body from deficiencies in calcium and vitamin D and consequent complications.

Calcium is mainly absorbed in the small intestine by active transport and passive diffusion. Approximately one-third of ingested calcium is absorbed, although it may vary depending on the form of the salt, dietary factors and the state of the small intestine. After absorption, calcium is eventually incorporated into bones and teeth with 99% of the amount of calcium present in the skeletal tissue of the body. The remaining is present in both the intra- and extracellular fluid. Approximately 47% of the total blood content of calcium is in the physiologically active ionized form with approximately 6% in complex with citrate, phosphate or other anions and the rest bound to proteins, primarily albumin. The absorption of calcium from calcium citrate is much higher than that of calcium carbonate^4^^,^ 5. In the present study, was found an improvement only in PTH with calcium supplementation, with both calcium carbonate and calcium citrate having positive effects.

Calcium can be bound to albumin (40%) and other anions (citrate and phosphate - 10%) and can be in the ionized form (50%)^6^. It functions in the permeability of cell membranes, muscle contraction and relaxation, nerve excitability, activation of enzymes and blood clotting. Its regulation involves some hormones such as vitamin D, PTH and calcitonin. Absorption occurs throughout the small intestine and jejunum and is influenced by several factors: pH, food intake (which may be a determinant factor for patients with RYGB, who have drastically lower protein intake), fat intake (also decreased among operated patients), amino acids, and intestinal motility^7^. That effervescent potassium calcium citrate was superior to citracal in conferring bioavailable calcium and suppressing parathyreoid hormone secretion^8^.

PTH is a hormone secreted by the parathyroid glands, which is controlled by calcium concentration. It has biological effects in three target organs, bones, kidneys and intestinal mucosa^9^. 

In extracellular metabolism, ionized calcium is metabolically better available and reflects the concentration of calcium. Both acidosis and alkalosis alter the binding capacity and amount of calcium. It acts physiologically with PTH and 1.25 (OH)_2_D_3_ 10 ^,^ 11.

A low concentration of circulating calcium leads to increased PTH and a reduction in bone mass, particularly a depletion of calcium and phosphorus, in attempt to increase blood calcium. At the same time, the kidneys increase the excretion of phosphorus and calcitriol and reduce calcium excretion^12^^,^ 13.

This whole mechanism causes secondary hyperparathyroidism which should be treated with calcium and vitamin D^13^. In the present study, this change in calcium excretion was not enough to cause osteopenia, osteomalacia or osteoporosis in the 20 participants. All received supplementation on time with either calcium carbonate or calcium citrate, reversing this complication after RYGB.

There were some limitations in this study. The participants underwent bone densitometry at various laboratories^14^. Protein intake was not fully investigated, but in a 24 h dietary record examined during nutritional consultation, it was possible to see the drastic decrease in protein intake as a whole and in many patients, particularly in the intake of protein rich in calcium, such as milk due to unwanted lactose intolerance, which can occur after RYGB.

It is interesting to note the importance of time of use of the calcium supplement, where calcium citrate or calcium carbonate, combined with vitamin D, for 60 days was sufficient to correct PTH values. What needs to be emphasized is that patients should use supplementation routinely and have regular blood tests to avoid hypervitaminosis and cardiac complications due to excess calcium. 

Supplementation is recommended in the immediate postoperative period to prevent the progressive loss of bone mass^15^^,^ 16. Many patients do not adhere to regular supplement use. They ignore the fact of intestinal malabsorption, where many important absorptive sites are lost, and furthermore hypochlorhydria can also compromise the absorption of calcium^17^. Idealy may be to start with 1000 mg calcium malat citrate and calcium carbonate, with 400UI vitamin D_3_ together after the surgery for prevent secondary hyperparathyroidism. 

Further studies are needed to compare citrate and carbonate salts for the treatment of secondary hyperparathyroidism after RYGB, including detailed monitoring of protein intake and urinary and fecal excretion.

## CONCLUSION

Both treatments with calcium malat citrate and calcium carbonate associated with vitamin D_3_ are efficient to correct the secondary hyperparathyroidism after the bariatric surgery. 
